# Pathogenic mechanisms of disease in idiopathic inflammatory myopathies: autoantibodies as clues

**DOI:** 10.3389/fimmu.2024.1439807

**Published:** 2024-08-30

**Authors:** Yuanhui Wu, Jiao Luo, Lihua Duan

**Affiliations:** ^1^ Jiangxi Province Key Laboratory of Immunity and Inflammation, Jiangxi Provincial People's Hospital, Nanchang, China; ^2^ Department of Rheumatology and Clinical Immunology, Jiangxi Provincial People’s Hospital, The First Affiliated Hospital of Nanchang Medical College, Nanchang, China; ^3^ JXHC Key Laboratory of Rheumatology and Immunology, Jiangxi Provincial People’s Hospital, Nanchang, China

**Keywords:** autoantibodies, idiopathic inflammatory myopathies, dermatomyositis, polymyositis, autoantibodies targeting aminoacyl-tRNA synthetases (ARS)

## Abstract

Idiopathic inflammatory myopathies (IIMs) encompass a spectrum of autoimmune diseases characterized by muscle inflammation and systemic involvement. This review aimed to synthesize current evidence on the clinical significance and pathogenic mechanisms underlying autoantibodies associated with IIMs. Autoantibodies targeting aminoacyl-tRNA synthetases (ARS) play a pivotal role in antisynthetase syndrome (ASS), highlighting associations with interstitial lung disease (ILD) and distinctive clinical features. Anti-Mi-2 antibodies in dermatomyositis (DM) are hallmarked by characteristic cutaneous manifestations and favorable prognostic outcomes. Conversely, anti-TIF1 antibodies are correlated with DM and a higher risk of malignancies, implicating CD8^+^ T cells in its pathogenesis. Anti-MDA5 antibodies signify clinically amyopathic DM (CADM) with severe ILD, linked to dysregulated neutrophil extracellular trap (NET) formation. In immune-mediated necrotizing myopathies (IMNMs), anti-SRP and anti-HMGCR antibodies induce complement-mediated myopathy, typically following statin exposure. Additionally, anti-TRIM72 antibodies emerge as potential diagnostic markers in IIMs. Anti-cN1A autoantibodies are linked to inclusion body myositis (IBM) and play a decisive role in muscle protein degradation. Meanwhile, anti-FHL1 autoantibodies are associated with severe disease manifestations and muscle damage, as established in experimental models. Anti-eIF3 autoantibodies, recently identified in polymyositis (PM) patients, are rarely detected (<1%) and associated with a favorable prognosis. Elucidating these autoantibodies is anticipated to not only assist in early diagnosis and disease stratification but also inform targeted therapeutic interventions, emphasizing the intricate interplay between autoimmunity, cellular dysfunction, and clinical outcomes in IIMs.

## Introduction

Idiopathic inflammatory myopathies (IIMs) are heterogeneous autoimmune diseases, including dermatomyositis (DM), polymyositis (PM), immune-mediated necrotizing myopathy (IMNM), inclusion body myositis (IBM), and anti-synthetase syndrome (ASS), that are characterized by inflammation of the proximal arm and leg muscles, as well as other organs, such as the skin, lung, and joint (1–4).

Autoantibodies are detectable in up to 80% of the patients with IIMs. They can be categorized into two major groups, namely myositis-specific autoantibodies (MSAs) and myositis-associated autoantibodies (MAAs), based on their specificity and clinical correlations. The former is typically characterized by a high level of specificity and holds significant value for both diagnosis and prognosis, particularly in categorizing patients into distinct or uniform subgroups for treatment purposes (2, 5–8). MSAs identified so far are autoantibodies against aminoacyl transfer RNA synthetases (ARS) including histidyl (Jo 1), alanyl (PL 12), threonyl (PL 7), glycyl (EJ) (9), isoleucyl (OJ), asparaginyl (KS), tyrosyl (Ha) and phenylalanyl (Zo) (10), signal recognition particle (SRP), melanoma differentiation-associated (MDA) 5/CADM 140, transcription intermediary factor 1 (TIF1), nuclear matrix protein (NXP) 2/MJ, Mi 2, 3-hydroxy-3-methylglutaryl-coA reductase (HMGCR) and small ubiquitin-like-modifier activating enzyme (SAE) (8, 11–13). MAAs are autoantibodies that are also present in other conditions that may culminate in myositis, such as systemic sclerosis (SSc) and systemic lupus erythematosus. MAAs include antibodies to SSA/Ro52, PM/Scl75, PM/Scl100, U1RNP, U1RNP, Ku and La (1, 6, 12, 14–21). The different types of MSAs and MAAs described in IIM have varying relevance for disease stratification, prognosis, and management.

The pathophysiology of IIMs is intricate and multifaceted and remains elusive. Its pathogenesis has been hypothesized to be influenced by genetic, environmental, and immunological factors. Notably, an increasing number of autoantibodies have been identified in recent years. Several autoantibodies play a clear pathogenic role in autoimmune disorders such as Grave’s disease, wherein thyrotropin receptor autoantibodies stimulate the overproduction of thyroid hormones, ultimately leading to hyperthyroidism. In myasthenia gravis, autoantibodies bind to acetylcholine receptors and inhibit the neurotransmitter acetylcholine from reaching muscle fibers, thereby inducing muscle weakness and fatigue (22). Nonetheless, the role of autoantibodies targeting endogenous antigens in disease pathogenesis or their presence as an epiphenomenon remains controversial (7, 23–26).

The specific clinical phenotypes associated with each MSA have raised questions on the role of these autoantibodies in the pathogenesis of IIM. Thus, this review examined the distinct clinical features associated with MSAs that potentially play a pathogenic role in the disease mechanisms. Thereafter, existing evidence from clinical longitudinal studies and experimental studies that might validate the pathogenic role of MSAs was outlined. However, the role of these antibodies in IIM is controversial. Our discussion focused on MSAs associated with the clinical subgroups of IIM and the possible pathogenic mechanism of autoantibody in myositis (**Table 1**). However, given the paucity of data suggesting that MAAs play a role in the pathogenesis of IIM, they were not comprehensively discussed in this review.

**Table 1:** MSAs in idiopathic inflammatory myopathies.

Antibody	Target antigen	Positivity rate	Clinical features	Rat model	Mechanism	Gene association
Anti-Jo-1	Histidyl-tRNA synthetase (HisRS)	15%–30%	muscle weakness, arthritis, Raynaud’s, mechanic’s hands, Gottron's sign, and/or interstitial lung disease (ILD), favorable outcome	Mice receiving IM injections of IFA emulsions containing the TLR7/8 agonist R848	Upregulation of genes associated with tumor necrosis factor (TNF)-α, triggering receptor expressed on myeloid cells-1 (TREM-1), CD25, and mitochondria pathways	HLA-DRB1*03
Anti-Mi-2	Component of the nucleosome-remodeling deacetylase complex consisting of histone deacetylase and nucleosome remodeling activities in an ATP-dependent manner	~ 10%	“Classical” form of DM with typical cutaneous lesions (Gottron's papules and sign and heliotrope exanthema) and muscle weakness. Low risk of ILD, cancer, favorable outcome	-	IFN1 pathway activation	HLA-DR7 HLA-DRB1*07DQA1*02DQB1*02 haplotype HLA-DRB1*07:01 HLA-DRB1*03:02
Anti-TIF1γ	Transcription intermediary factor 1	5–7%	Malignancy, classical DM skin rash, and muscle weakness	TIF1γ-induced myositis (TIM) mice	-	HLA-A*11: 01,HLA-B*44: 03 HLA-DQA1*03:01
Anti-MDA5	Melanoma differentiation-associated gene 5	8%-20%	Rapidly progressive interstitial lung disease, arthritis, and severe cutaneous vasculopathy (skin ulceration, tender palmar papules, or both)	Murine model of ILD mediated by autoimmunity against MDA5 mirroring the severe and rapid progression of ILD.	Type I IFN pathway, NF-κB pathway	HLA-DRB1*0101, *0405, *1201, *0901, *0401, and *1202
Anti-SRP	Signal recognition particle	~ 10%	Muscle atrophy, severe weakness (Anti-SRP myopathy patients are more likely to experience severe weakness and extra-muscular features)	Passive transfer of IgG from anti-SRP positive patients was performed in C57/Bl6 or Rag2-deficient or Complement 3 deficient mice.	Activation of the classical pathway of complement Transcription factors TRIM63/MURF1 and MAFbx, which are involved in the atrophy pathway	HLA-DQA1*01:04 HLA-DQA1*01:02
Anti-HMGCR	Hydroxy-3-methylglutaryl-CoA reductase	~ 10%	Necrotizing myopathy, statin-induced myopathy	Passive transfer of IgG from anti-HMGCR positive patients was performed in C57/ Bl6 or Rag2 deficient or Complement 3 deficient mice.	-	HLA-DRB1*11:01 HLA-DRB1*07:01
Anti-TRIM72	Muscle-enriched membrane repair protein	–	-	Trim72–/–mice	-	-
Anti-cN1A	Cytosolic 5'-nucleotidase 1A	33% of IBM and 4.3% of PM/DM patients	Bulbar involvement, high mortality rates associated with lung complications, older age at disease onset, or no clinical association	Anti-cN1A injected mice	-	HLA-B8-DR3
Anti-FHL1	Four and a half LIM domain protein 1	14%-25%	Pronounced muscle fiber damage, muscle atrophy, vasculitis, and dysphagia	MHC class I transgenic mice were immunized with FHL-1 protein and adjuvant	-	HLA alleles DRB1*07 and DRB1*15
Anti-eIF3	Eukaryotic initiation factor 3	<1%, 0.44% of PM	Highly elevated creatine kinase (CK) levels, proximal muscle weakness, favorable outcome	eIF3f gene knockout mice	mTORC1 pathway	-

## Anti-aminoacyl-tRNA synthetases autoantibodies

As is well documented, aminoacyl-transfer RNA synthetases (ARSs) are enzymes responsible for the first step of protein synthesis, attaching amino acids to their corresponding cognate transfer RNA (tRNA) sequences (17, 21, 27). Autoantibodies targeting ARSs, the most frequent MSAs detected in patients with IIMs, are associated with a distinct clinical phenotype termed “antisynthetase syndrome (ASS)”, characterized by the presence of one anti-ARS antibody plus one or more of the following manifestations: interstitial lung disease (ILD), myositis, arthritis, Raynaud’s phenomenon, fever, or mechanic’s hands (28, 29). Notably, ARS has been detected in 25%-35% of patients with ASS. To date, autoantibodies targeting 8 out of 21 ARSs have been identified and associated with ASS. While other less common antisynthetase autoantibodies have been reported in the literature, they are infrequently studied in the clinical setting. Known targets are histidyl (Jo1), threonyl (PL-7), alanyl (PL12), glycyl (EJ), isoleucyl (OJ), tyrosyl (Ha/YRS), asparagyl (KS), phenylalanyl (Zo), lysyl (SC), glutaminyl (JS), and tryptophanyl (WRS) tRNA synthetases (30–33).

## Anti-Jo-1

Anti-Jo1 [anti-histidyl-tRNA synthetase (HisRS)] is the most common MSA that is present in 20-30% of IIM patients (2, 21, 34). A meta-analysis involving 27 studies investigating the clinical characteristics of ASA concluded that anti-Jo1 is linked to a higher risk of mechanic’s hands, arthritis, and myositis. Patients with anti-Jo-1 positive antibodies may experience Raynaud’s phenomenon before developing myositis (27). Worldwide epidemiological studies have documented that up to 90% of patients with anti-Jo1 autoantibodies develop ILD (4, 9, 26, 31, 34, 35). In the American and European Network of Antisynthetase Syndrome (AENEAS) cohort study recruiting anti-Jo1-positive patients, ILD was present in 50% of cases at disease onset and in 84% of patients after an 80-month follow-up period (19, 28). Moreover, anti-Jo-1-positive ASA patients have higher 5- and 10-year survival rates compared to non-Jo-1 patients, potentially ascribed to earlier diagnosis facilitated by the increased availability of anti-Jo1 testing (33). MHC class II alleles have been reported to be associated with certain MSAs, such as anti-Jo1 autoantibodies and the HLA 8.1 ancestral haplotype containing HLA-DRB1*03:01 (36). In addition, environmental factors can also induce ASS. Indeed, a strong association was noted between anti-Jo1 antibodies and HLA-DRB1*03:01 is strongest in patients with a smoking history.

Several experiments in human sera and passive transfer to mice indicate an immune response toward HisRS. *In vitro* studies have demonstrated that the N-terminal domain serves as a chemoattractant for naïve lymphocytes and immature dendritic cells through interaction with CCR5 (37). However, it is unknown whether or how the anti-Jo-1 antibodies regulate the chemokine activity of Jo-1. Instead, anti-Jo1 can form an immune complex (IC) that stimulates the synthesis and release of type I interferon by plasmacytoid dendritic cells (38). Furthermore, T-cell stimulation assays using a peptide from the HisRS N-terminal domain elicited an inflammatory response in blood and bronchoalveolar T-cells (29, 39, 40). Additionally, germinal center-like structures were identified in the lung tissue of anti-Jo1-positive patients, supporting the hypothesis of the lungs as a potential site for immune activation and production of anti-Jo1 autoantibodies. Numerous mice experiments have been performed to explore the role of HisRS in the pathogenesis of the disease. Initially, mice immunized with HisRS and adjuvant generated anti-HisRS antibodies but did not develop myositis, implying a species-specific antigenic immune response. In contrast, cDNA inoculation of human HisRS in mice induced inflammatory infiltrates in muscle along with detectable anti-HisRS antibodies, especially when using a truncated gene containing the N-terminal domain of the HisRS coding region, indicating different immune responses upon antigen presentation. Moreover, in an adjuvant-based model, the administration of murine N-terminal HisRS was capable of breaking tolerance and promoting T-cell proliferation and epitope spreading, causing muscle and lung inflammation similar to the antisynthetase syndrome (39). Histological studies of muscle tissues revealed diverse infiltration patterns, with perimysial/epimysial inflammation in a perivascular distribution, endomysial inflammation, and muscle fiber invasion/degeneration. High levels of anti-HisRS antibodies in bronchoalveolar lavage fluid and serum were detected in this mouse model (39). Furthermore, in an antigen-driven model, several strains of mice immunized with murine N-terminal HisRS displayed early T-cell infiltration in muscle and IgG class-switched autoantibody responses that persisted for at least 7 weeks (39). Further mouse experiments have explored the role of the MyD88 signaling pathways and highlighted the contributions of TLR2 and TLR4 (41). C3H/HeJ (TLR4-KO) mice deficient in anti-HisRS antibodies exhibited muscle inflammation induced by immunization with HisRS. These findings signal a key role for innate immune responses, but not HisRS-specific autoimmunity, in a HisRS-induced model of myositis (16). These findings collectively suggest that HisRS may induce myositis, but these models do not fully unravel the link between innate and adaptive immune responses. Taken together, additional *in vitro* and *in vivo* experiments are warranted to assess the mechanism of anti-Jo-1 antibodies in myositis.

## Anti-Mi-2 autoantibody

Anti-Mi-2 is a dermatomyositis-specific autoantibody that targets antigens such as Mi-2a (240 kDa) and Mi-2b (218 kDa), thereby forming a protein complex with histone deacetylases, referred to as the nucleosome remodeling deacetylase (NuRD) complex. This autoantibody has been detected in patients with hallmark cutaneous DM lesions, including Gottron’s papules, heliotrope rash, cuticular overgrowth, and rashes on the neck and upper back or shoulders (V neck and shawl sign) (2, 6, 42, 43). The prevalence of anti-Mi-2 autoantibodies in DM patients varies from 5-10% in adults and 4-10% in juveniles. Furthermore, anti-Mi-2-positive patients typically have relatively mild muscle involvement, fewer complications such as ILD or cardiac disease, and are generally responsive to treatment with a favorable prognosis (43, 44).

Previous studies have established that anti-Mi-2 DM is associated with prominent pathological muscle involvement hallmarked by marked inflammatory cell infiltration. Tanboon et al. (45) concluded that anti-Mi-2 DM patients had a higher level of CD3^-^ and CD20^+^ cell infiltration in the endomysium and CD68+ cell infiltration in the perimysium compared to non-Mi-2 DM patients. Anti-Mi-2 DM was also more frequently associated with CD20^+^ cell aggregation and ACP/CD68 cell infiltration in non-necrotic fibers. These findings, together with higher CK levels, may account for the more severe clinical muscle involvement observed in anti-Mi-2 DM patients (46).

Meanwhile, a higher level of CD68^+^ cell infiltration in the perimysium may explain the more frequent perimysial connective tissue alkaline phosphatase (ALP) activity in anti-Mi-2 DM patients. Considering that the expression of tissue nonspecific alkaline phosphatases can be up-regulated by cytokines such as tumor necrosis factor-alpha (TNF-α) and interleukin-1 beta (IL-1β), a subset of CD68^+^ cells in the perimysium has been speculated to secrete these cytokines, resulting in increased perimysial connective tissue ALP activity in anti-Mi-2 DM patients. On the contrary, perimysial connective tissue fragmentation could be attributed to inflammation and edema, which may account for the comparable percentages of perimysial connective tissue fragmentation between the anti-Mi-2 and non-Mi-2 DM groups.

In individuals with myositis autoantibodies, antibodies accumulate within myofibers in the same subcellular compartment as their corresponding autoantigens. Each autoantibody exerted effects that were in line with the malfunction of its corresponding autoantigen, such as internalization of antibodies from anti-Mi2 patients causing the derepression of Mi2/NURD-regulated genes (47). The study further evinced that anti-Mi2 autoantibodies exerted pathogenic effects by infiltrating damaged myofibers and inhibiting the CHD4/NuRD complex (48).

Besides, several studies have examined the relationship between exposure to ultraviolet (UV) light and the risk of anti-Mi-2-positive dermatomyositis. Love et al. determined that UV radiation intensity was correlated with the incidence of DM and anti-Mi-2-positivity (30, 49, 50). Burd et al. described that UV light upregulated Mi-2 expression in human keratinocytes (51). Given the relationship between anti-Mi-2 and HLA-DRB1*07DQA1*02DQB1*02 haplotype, HLA-DRB1*07:01 and HLA-DRB1*03:02, genetic background, as well as environmental factors, may influence the development of anti-Mi-2-positive DM.

## Anti-TIF1 autoantibody

Anti-TIF1 targets TIF1γ of 155 kDa with or without TIF1α, formerly referred to as anti-p155 (52) and as anti-155/140 (53). Anti-TIF1 autoantibodies are specifically present in DM (15-20% of adult DM and 20% of juvenile DM cases). Anti-TIF1γ has a positive association with malignancy, especially when co-occurring with anti-TIF1α and a negative association with ILD (7). A meta-analysis involving 6 studies and 312 adult DM patients reported that the pooled sensitivity, specificity, and diagnostic OR of anti-p155 for the diagnosis of cancer-associated DM was 78% (95%, CI 45%–94%), 89% (95%, CI 82%–93%), and 27.26% (95%, CI 6.59%–112.82%), respectively (2, 49, 54, 55). Lastly, genetic susceptibility studies established that anti-TIF1 antibody was associated with HLA-DQA1*03:01 (52).

Anti-TIF1γ antibody-positive DM is linked to a higher risk of malignancy, especially in older patients (56). The presence of TIF1γ is significant in both cancerous tissues and during pregnancy, suggesting that it serves as a potential trigger for autoimmunity against TIF1γ (57). TIF1γ is frequently mutated or over-expressed in tumors and is over-expressed in embryonic and mammary epithelial cells during pregnancy. Therefore, cancer and pregnancy have been postulated to trigger autoimmunity against TIF1γ, which, in turn, contributes to the development of myositis. Konishi et al. established a TIF1γ-induced myositis model (TIM) in B6 mice via weekly subcutaneous injections of recombinant human TIF1γ protein emulsified in CFA four times, along with an intraperitoneal injection of pertussis toxin (PT) (16). As anticipated, the immunized mice developed TIF1γ-specific T cells and anti-human and murine TIF1γ antibodies, resulting in myositis in the hamstrings and quadriceps two weeks after the last immunization. Histological studies revealed atrophy and necrosis of muscle fibers, accompanied by infiltrating mononuclear cells in the perifascicular and endomysial sites of muscle tissues. Immunohistochemistry assays illustrated that CD8+ T cells predominantly infiltrated and adhered to muscle fibers, which upregulated the expression of MHC class I and type I IFN-responsive molecule Mx1. Beta 2 microglobulin-KO mice lacking MHC class I expression, perforin-KO mice, and anti-CD8 depleting antibody-treated mice rarely develop TIM (58). Meanwhile, adoptive transfer experiments demonstrated that CD8+ T cells derived from TIM mice could induce myositis in recipient B6 mice, whereas CD4+ T cells did not exert this effect. These findings identify CD8+ T cells as the primary pathogenic cells in TIM. In contrast, μMT mice, which completely lack B-cell lineages, developed myositis, whereas the adoptive transfer of IgGs collected from TIM mice failed to induce myositis in recipient mice. These results collectively indicate that B cells and autoantibodies are not essential for developing TIM. In other words, anti-TIF1γ antibodies detected in patients with DM may be a diagnostic biomarker but not a direct pathogenic factor. Accordingly, TIM, which is dependent on autoimmunity against TIF1γ, was mediated by TIF1γ-specific CD8+ T cells but not TIF1γ-specific CD4+ T cells, B cells, and autoantibodies.

The study evinced that B cells and autoantibodies are not essential for the development of TIF1γ-induced myositis (TIM), establishing that CD8+ T cells play a primary pathogenic role. Thus, the presence of anti-TIF1γ antibodies in patients with DM primarily serves as a diagnostic biomarker rather than a direct cause of the disease. The involvement of type I interferons in the pathogenesis of TIM and the effectiveness of tofacitinib treatment highlight potential therapeutic targets. The TIM model thus provides a robust framework for studying DM and pioneering targeted treatments.

## Anti-MDA5 autoantibody

Anti-MDA5, reported to be a specific autoantibody for clinically amyopathic DM (CADM), was first named anti-CADM-140 in 2005 (59, 60). Subsequently, the target autoantigen was identified as melanoma differentiation-associated gene 5 (MDA5), also known as interferon-induced with helicase C domain protein 1 (IFIH1). Of note, MDA5 is a cytoplasmic retinoic acid-inducible gene-I (RIG-I)-like receptor that, upon the recognition of viral RNA, up-regulates the expression of type 1 interferon and other inflammatory cytokines (2, 49, 61).

Furthermore, the anti-MDA5 autoantibody is detected in 20-50% of adult DM patients (including CADM) and is associated with relatively lower creatine kinase (CK) levels, a high frequency of ILD (90-95%), especially rapidly progressive ILD (RP-ILD) (50-80%), and poor prognosis due to respiratory failure (62–65). However, lower frequencies of the antibody and RP-ILD were noted in American and European cohorts (49). Such clinical discrepancies might be explained by differences in ethnicity or environmental background. Anti-MDA5 antibody is also associated with HLA-DRB1*01:01/*04:05 (66).

Increased serum IL-6, IL-8, and IL-10 levels were associated with RP-ILD in PM/DM patients, whilst high concentrations of IFN-α and soluble CD163 and the upregulation of IFN-inducible genes have been detected in anti-MDA5-positive patients. These findings suggest the upregulation of the type 1 IFN system through the activation of monocytes, macrophages, or other immunocompetent cells in the pathophysiology of anti-MDA5-positive DM with RP-ILD (59). Depending on disease severity, autoAbs profiles differ among anti-MDA5+ patients, with autoAbs from B cells of patients directly stimulating IFN-gamma production in the peripheral blood. Mounting evidence indicates that dysregulated NET formation participates in the pathogenic process of IIM, particularly in anti-MDA5-positive disease (67, 68). Circulating NET levels were increased while the plasma DNase I activity was impaired, resulting in the failure to degrade the aberrant NETs in patients with anti-MDA5 autoantibodies, especially in those with ILD. Enhanced NET formation was observed in affected organs, including the skin, muscle, and lungs, of anti-MDA5-positive patients but not in those with MSA-negative IIM (67).

A recent study also delineated that anti-MDA5 autoantibodies isolated from patients significantly enhanced NET formation in neutrophils isolated from healthy controls compared with control IgG. Furthermore, NETs purified from IIM neutrophils have been observed to induce myotube damage. Another study described that peripheral NET levels were associated with calcinosis, ICs, and IL-8 levels in patients with JDM (49, 69). In particular, JDM patients with anti-MDA5 autoantibodies exhibited impaired NET clearance. Indeed, anti-MDA5 autoantibodies play a critical role in inducing NET formation. The study emphasizes the need for further investigation into the mechanisms underlying NET dysregulation and its contributions to autoimmune responses and tissue damage in IIM.

In addition, an earlier study found that a murine model of ILD mediated by autoimmunity against MDA5 mirrors the severe and rapid progression of ILD observed in patients with anti-MDA5 antibody-positive DM. Key findings highlighted the vital role of CD4+ T cells and IL-6 in the development and severity of fibrotic ILD. The results suggest that targeting IL-6 could be a potential therapeutic approach for the management of ILD in patients with anti-MDA5 antibody-positive DM (70). Animal models are valuable for studying disease mechanisms, given that they allow for controlled experimentation and manipulation of specific immune components. The evidence from these models convincingly unveiled that T cells can induce disease independently of autoantibodies, emphasizing their role in disease pathogenesis. Despite the compelling evidence from animal models, there are limitations that cannot be overlooked. Animal models may not fully reflect the complexity of human disease, and findings in animals may not always translate directly to humans. Besides, the presence of autoantibodies in patients with DM is associated with specific clinical features and outcomes, suggesting they still play a role in the disease, albeit not as primary inducers.

## Anti-SRP and anti-HMGCR autoantibodies

Anti-signal recognition particle (anti-SRP) antibody was initially identified in a subgroup of polymyositis patients in 1986 (71). In 2002, muscle the presence of necrotic muscle fibers without significant muscle inflammation was detected in biopsies from anti-SRP antibody-positive patients (72). In 2003, a group of immune-mediated necrotizing myopathies (IMNMs) was recognized for the first time as a separate entity, based on pathological criteria showing predominant muscle fiber necrosis with no or mild muscle infiltrates, and these patients generally have a poor prognosis (73–75). A novel myositis-specific antibody targeting the hydroxy-3-methylglutaryl-CoA reductase (HMGCR) protein was discovered in a subset of IMNM patients thereafter (6, 7, 76–78).

Anti-SRP patients exhibit more severe muscle weakness and atrophy with substantial muscle damage in magnetic resonance imaging studies (79–81). Anti-SRP is detected in 2% of patients with adult dermatomyositis. Moreover, approximately 10-20% of anti-SRP patients develop extramuscular symptoms, especially ILD (80). Anti-SRP was associated with HLA-DQA1*01:04 and HLA-DQA1*01:02. Conversely, anti-HMGCR patients are often linked to statin exposure (82, 83) and were detected in 6% of adult dermatomyositis cases. Importantly, it was associated with HLA-DRB1*11:01 and HLA-DRB1*07:01. For both autoantibodies, a high correlation between CK levels and MAC (C5b-9) deposits with the percentage of myofiber necrosis was reported (r=0.6, p < 0.01 and r=0.4, p < 0.01, respectively) (84). The titer of these auto-antibodies correlates with disease activity in anti-SRP and anti-HMGCR-positive patients (49). Histopathological analysis of muscle biopsies from anti-HMGCR-positive patients revealed muscle fiber degeneration and regeneration, as well as up-regulation of MHC-I on occasional non-necrotic muscle fibers with rare or absent inflammatory infiltrates (22, 76, 83, 85, 86). Furthermore, necrotic fibers were largely associated with CD68^+^ macrophage infiltration and membrane attack complex (MAC) deposition on scattered non-necrotic fibers, suggesting the presence of an antibody-dependent cell-mediated toxicity pathway (87).

These autoantibodies were associated with an increased secretion of proinflammatory cytokines (IL-6 and TNF), reduction in the levels of anti-inflammatory cytokines (IL-4 and IL-13), production of reactive oxygen species, and upregulation of genes encoding atrophic factors. This decrease of IL-4 and IL-13 suppressed myotube formation by impairing myoblasts fusion. In *in vitro* experiments on myotubes, incubation with anti-SRP antibodies, anti-HMGCR antibodies, or total IgG from patients’ plasmapheresis induced atrophy and was associated with increased expression of the transcription factors TRIM63/MURF1 and MAFbx, which are involved in the atrophy pathway. *In vitro* experiments showed that purified anti-SRP and anti-HMGCR autoantibodies could recognize their cognate autoantigens and activate the classical complement pathway (87). Experimental studies in animal models further support the pathogenic role of the anti-SRP and anti-HMGCR autoantibodies (88, 89). Purified IgG from anti-HMGCR and anti-SRP-positive patients was injected in mice, provoking muscle deficiency and myofiber necrosis, similar to human disease (89). Interestingly, muscle deficiency tends to be more severe in mice receiving IgGs from anti-SRP antibody-positive patients compared to those receiving IgGs from anti-HMGCR antibody-positive patients. Immunization with SRP and HMGCR protein drove the production of specific antibodies, indicating a pathogenic association with these proteins. Myopathy in IMNM was alleviated in IgG-transferred complement C3-KO mice, whereas supplementation with human complement reversed this effect (16). The study implied that patient-derived anti-SRP and anti-HMGCR antibodies are pathogenic toward muscles *in vivo* through a complement-mediated mechanism. However, treatment with a C5 complement inhibitor was not effective in anti-SRP-positive and anti-HMGCR-positive patients (90).

The research highlights the dual diagnostic and pathogenic role of anti-SRP and anti-HMGCR autoantibodies in IMNMs, as well as their potential impact on muscle pathology through cytokine modulation, macrophage infiltration, and complement activation.

## Anti-TRIM72 autoantibody

Several tripartite motif (TRIM) family proteins (Ro52, TIF1α, TIF1β, and TIF1γ) are well-established autoantigens associated with IIM (91–93). a novel TRIM family protein termed TRIM72 (also known as MG53) and its function were identified in IIM. TRIM72 is an integral component of the sarcolemmal repair process in striated muscle (94–96). ELISA analysis uncovered elevated TRIM72 autoantibody levels in IIM, with 11.5% of DM sera and 11.8% of PM sera tested presenting with high levels of anti-TRIM72 (97). Trim72^–/–^ mice develop significant skeletal muscle myopathy and cardiovascular defects due to defective sarcolemmal repair (98–100). In an adoptive transfer mouse model, sarcolemmal resealing defects were detected at 1 and 4 weeks, indicating that in a Treg-deficient/dysfunctional environment, a marginal increase in anti-TRIM72 levels is correlated with reduced sarcolemmal resealing capacity. Additionally, exogenous delivery of a polyclonal antibody against TRIM72 significantly reduced sarcolemmal resealing capacity in flexor digitorum brevis (FDB) muscles from healthy C57BL mice regardless of anti-TRIM72 levels. Overall, the findings suggest that a defect in sarcolemmal resealing may precede skeletal muscle degeneration and inflammation associated with IIM (97). Taken together, these findings highlight the essential role of TRIM72 in muscle membrane repair and its potential involvement in the pathogenesis of IIM through autoantibody production and impaired membrane resealing.

## Anti-cN1A autoantibody

Anti-cytosolic 5’- nucleotidase 1A (cN1A) was described in 2011 by Salajegheh et al. as an autoantibody against a 43 kDa protein associated with inclusion body myositis (IBM) (1, 7, 101, 102). The corresponding autoantigen was identified as cN1A expressed in skeletal muscle (103). This autoantigen is implicated in the hydrolysis of adenosine monophosphate, leading to physiological energy homeostasis, metabolic regulation, and cell replication (103, 104). Anti-cN1A is present in about 33-34% of IBM, 4-5% of PM, and 3-4% of DM cases. Considering that Herbert et al. concluded that this autoantibody is present in 36% of patients with Sjogren’s syndrome and 20% of SLE cases, the specificity of this autoantibody for myositis is likely low (105, 106). it might assist in differentiating between myositis subgroups (49, 106–108).

A recent study demonstrated the pathogenic role of anti-cN1A in IBM both *in vivo* and *in vitro* using a passive immunization model. The anti-cN1A autoantibody potentially affects protein degradation in myofibers (109). Several experimental *in vivo* and *in vitro* passive immunization studies in mice were performed. The results similarly identified that anti-cN1A antibodies could impact muscle protein degradation and fiber size with small angulated fibers in mice injected with anti-cN1A-positive sIBM IgG. However, these experiments failed to demonstrate changes in motor activities. The findings conjointly indicate that anti-cN1A autoantibodies play a role in the pathology of IBM by altering muscle protein degradation and muscle fiber morphology. Nonetheless, the lack of changes in observed motor activity suggests further research is warranted to fully elucidate the clinical impact of these autoantibodies.

## Anti-FHL1 autoantibody

Four-and-a-half-LIM-domain 1 (FHL1) is a muscle-specific antigen abundantly expressed in skeletal and cardiac muscle. Mutations in the FHL1 gene have been detected in diverse X-linked myopathies (1, 110, 111). The prevalence of anti-FHL1 autoantibodies has been reported to range between 14 and 25% of IIM patients and has been associated with poor prognostic characteristics such as pronounced muscle fiber damage, muscle atrophy, vasculitis, and dysphagia. In an independent cohort, anti-FHL1 autoantibody frequency was higher in PM and IBM patients and was frequently observed in those with MSA-negative IIM.

To explore the role of FHL1, MHC class I transgenic mice were immunized with FHL1 protein and adjuvant, which resulted in muscle inflammation, weakness, and weight loss nine weeks after the immunization in double-transgenic mice but not in single-transgenic mice. The presence of anti-FHL1 autoantibody was detected in both immunized mice groups. HT mice had significantly lower survival rates, and histopathological examination exposed prominent muscle damage with IgM depositions, suggesting a link between anti-FHL1 responses and muscle damage. Anti-FHL1 autoantibodies serve as markers for more severe disease in IIM, and the experimental data reinforce their pathogenic role, underscoring the importance of FHL1 in the disease mechanism.

In total, the presence of anti-FHL1 autoantibodies in IIM is indicative of more severe disease manifestations. The experimental evidence from transgenic mice corroborates the pathogenic role of anti-FHL1 responses, emphasizing the role of FHL1 in muscle damage and inflammation in IIM patients.

## Anti-eIF3 autoantibody

The anti-eukaryotic initiation factor 3 (eIF3) autoantibody was recently identified in the sera of three Caucasian patients with PM. Noteworthily, the level of this autoantibody was low (<1%) and was associated with a good prognosis and a favorable response to treatment (1). The depletion of eIF3 in mouse models was associated with reduced skeletal muscle mass, indicating that this protein might play a paramount role in muscle growth and skeletal muscle homeostasis (1, 112) However, these associations remain to be investigated in further studies.

## Conclusion

This review summarizes the classical and novel MSAs in IIM identified over the past years. These autoantibodies are preferentially expressed in disease-associated tissues and play a major role in disease initiation and propagation. For some of the MSAs, experimental data support their potential role in the pathogenesis of IIM. Understanding the functional role of these autoantigens and their corresponding autoantibodies in disease initiation, propagation, and expression is fundamental for providing further insights into pathogenic pathways, which in turn may facilitate the development of new therapeutic targets.
